# Congenital Hereditary Atonic Dyspepsia

**Published:** 1887-01

**Authors:** R. Walker Beers


### *CONGENITAL HEREDITARY ATONIC DYSPEPSIA.*

During a practice of twenty years, I have prescribed Lactopeptine to patients of all ages, and have never been disappointed in its action when indicated. But I desire to speak in particular of its action in a case of congenital hereditary atonic dyspepsia: in an infant, to whom I began to administer this remedy on the third day after birth. Mrs. H. L. S., Langside, Miss., was delivered of a male child in whom there was manifested well-marked symptoms of atonic dyspepsia. The mother had been a victim of dyspepsia from girlhood, and had inherited the malady from her mother.

The infant was put to the breast a few hours after birth, and nursed readily; but almost immediately rejected the milk. Repeated trials all resulted in vomiting, followed by exhaustion. Other articles of food were tried, including cow's milk, etc., without improvement. The child was in great danger of starvation. On the third day, I began the administration of Lactopeptine. The effect was immediate and almost miraculous. I ordered one-sixteenth of the adult dose to be dissolved in about two ounces of breast milk (drawn from a robust, healthy wet-nurse,) and administered every two and a half hours. There was no more rejection of milk—except the usual vomiting of curdled milk, to relieve the crowded state of the stomach, which occurred occasionally, after the first ten days. Condensed milk, cow's milk (properly diluted and sweetened), Mellin's food, boiled bread, (pap,) were, after a while, substituted for breast milk, but always with Lactopeptine. A steady improvement was manifest from the beginning, and kept up during the first dentition, which process was gone through with in a most satisfactory manner. No untoward diarrhœa or intestinal disturbance characterized this period, and, at ten months, the child was virtually cured of its dyspepsia, and could eat and digest ordinary food such as children of that age may do in good health. The parents of the child believe firmly (as I do) that Lactopeptine saved their infant.

In cholera infantum, in diarrhœa, and in all of the disturbances of the alimentary canal during dentition and early infant life, I find Lactopeptine an ever-effective and reliable remedy. In adult dyspepsia, all are now familiar with its beneficial effects; but I should be glad if the profession would be induced to try it in the vomitings, diarrhœas and dyspepsias of infancy. I recall several babies whose lives, I believe, I could have saved had I known, ten years ago, what I do now of the ready adaptability of Lactopeptine to infants, ailments.—*R. Walker Beers, M. D., in the Medical Brief.*

